# Efficacy of extracting and preventively intervening late-stage older adults who are at high risk for spending high medical costs by using the health check-up system in Japan: a pilot study

**DOI:** 10.3389/fpubh.2024.1434800

**Published:** 2024-11-27

**Authors:** Kana Kazawa, Madoka Kawai, Michiko Moriyama

**Affiliations:** ^1^Division of Nursing Science, Graduate School of Biomedical and Health Sciences, Hiroshima University, Hiroshima, Japan; ^2^Faculty of Health Sciences, Okayama University, Okayama, Japan

**Keywords:** older adults, health checkups, health risk, hospitalization, education

## Abstract

**Objectives:**

In Japan, the seven diseases (femur fracture, cerebral infarction, chronic renal failure, heart failure, dementia, pneumonia, and chronic obstructive pulmonary disease) are the top causes of inpatient medical costs among the late-stage older adults aged 75 years and over. This pilot study was conducted with the following two objectives; (1) to examine the proportion of risks of onset and severity of seven diseases among the late-stage older adults, and (2) to examine the efficacy of interventions focusing on the prevention of unplanned hospitalization.

**Methods:**

Participants were 45,233 older adults aged 75 and over living in Kure City, Japan. In addition to the government-mandated health checkup items, the Intervention group underwent additional risk screening tests included questionnaires, physical examinations, blood tests, and educational guidance by nurses. The efficacy of the intervention was examined whether there were differences in the number of hospitalizations, the use of emergency and critical care, and the incidence of hemodialysis induction between the Intervention and control groups (Usual Health Checkup group and No Health Checkup group) for the 2 years.

**Results:**

There were 485 participants in the Intervention group, 1,067 in the Usual Health Checkup group, and 43,712 in the No Health Checkup group. As the risks of seven diseases in the Intervention group, the largest proportion of deviations occurred for systolic blood pressure (63.3%), estimated salt intake (60.3%), and low-density lipoprotein cholesterol (51.5%). Estimated glomerular filtration rate deviated in 41.0%, N-terminal pro b-type natriuretic peptide in 37.9%. 7.5% scored <2 points on the Mini-Cog©, and 9.1% performed the Timed Up and Go test in >12 s. The incidence of hospitalization due to any of the seven diseases was significantly higher in the No Health Checkup group (*p* < 0.001). There were no differences among the three groups in the use of emergency and critical care or the introduction of hemodialysis.

**Conclusion:**

This study revealed that additional health checkup tests and intervention methods could be prevented hospitalization among the adults of 75 years and older. It is necessary to make health checkups and follow-ups more accessible those are already available within the existing health system in Japan.

## 1 Introduction

As the global population ages (1), especially in developed countries, increasing healthcare costs are a challenge. Healthcare reforms have been implemented in many countries to achieve sustainable healthcare systems (2–5). Globally, the population of Japan comprises the highest percentage of individuals aged 65 years and older (1). In 2021, this percentage was 28.9%, while that of individuals aged 75 years and older was 14.9%, indicating that Japan will continue to have a super-aged society (6). According to national statistics, medical costs increase with age. Average medical costs are reported to be 4.0 and 4.9 times higher for individuals aged 65 years and older and those aged 75 years and older, respectively, compared with those for individuals aged 64 years and younger (7). Therefore, the use of medical resources by older adults tends to increase with age. However, several previous studies have noted that not all older adults utilize the same amount of resources, and have recommended the identification of those with high health risks requiring early intervention (8–10). The healthcare cost analysis conducted in our previous study revealed that approximately 40% of adults aged over 75 years spent more than 80% of their total healthcare costs (11). Additionally, ours and other studies revealed that older adults with higher healthcare costs tended to use more emergency and critical care medicine and require longer hospitalization (11–13). Therefore, interventions aimed at avoiding unplanned hospitalizations are essential for older adults with high health risks. Furthermore, medically dependent older adults require daily care caused by aging and disease as well as disease management. Thus, early identification of older adults with high health risks, providing preventive education, recommendations for medical examinations, and the introduction of services as needed are highly significant in terms of stabilizing their health status, maintaining their quality of life, and ultimately reducing healthcare costs. In particular, the percentage of adults aged 75 years and older who require daily care has increased dramatically to 31.9% (4.3% for those aged 74 years and younger) (6). This finding corroborates the need to focus on this age group.

In Japan, the following seven diseases are the top causes of inpatient medical costs among adults aged 75 years and older: femur fracture, cerebral infarction, chronic renal failure (CRF), heart failure (HF), dementia, pneumonia, and chronic obstructive pulmonary disease (COPD). The development or progression of these diseases can be avoided through proactive interventions, such as health screening and education and referral to appropriate services (11). However, the current community-based health checkup for adults aged 75 years and older in Japan consists of items focused on metabolic syndrome, not seven diseases (14). In advanced age group, screening for risk of high-frequency diseases, early detection, and early intervention will contribute to improve disease management and their quality of life as well as reduce the continually increasing healthcare costs.

Therefore, we collaboratively worked with the municipal government who was in charge of the health checkup, and added screening tests to this usual health checkup for community-dwelling individuals aged 75 years and older to enable the early detection of the risk for developing the seven diseases that are likely to lead to hospitalization, and the risk of serious illness in those who already have one or more of these conditions as a pilot trial. As an intervention based on the results of the medical checkups, nurses explained the results, provided self-management education, and, if necessary, recommended medical examinations and referrals to appropriate services.

This study aimed to (1) examine the rate of risk of onset and severity of seven diseases that were the leading causes of inpatient medical costs among adults aged 75 years and older and (2) examine the efficacy of interventions based on the results of health checkups, with a focus on preventing unplanned hospitalization. By clarifying the usefulness of screening and intervention according to the health risk characteristics of adults aged 75 years and older, this study will propose to the government the introduction of new health checkup tests that take health risk into the consideration and will lead to the consideration of health support method for municipal governments.

## 2 Materials and method

### 2.1 Study design

The study was a non-randomized controlled trial. Municipal governments as medical insurers in Japan recommend that insured individuals undergo health checkups; this study was conducted as part of the healthcare project by the medical insurer.

First, we clarified the risk of onset and severity of seven diseases that are likely to lead to hospitalization in older adults (frequency of the occurrence of deviations from normal values). Second, we examined whether additional health checkup tests and interventions by nurses can improve health outcomes.

### 2.2 Participants

Japan practices a universal medical care insurance system, and citizens are insured by some type of medical insurer. Medical Insurers encourage insured persons to undergo annual health checkups that comprehensively examine their health status regardless of whether the person has any diseases or not (15).

#### 2.2.1 Intervention group

The participants were older adults residing in Kure City, Hiroshima Prefecture, Japan, who met the following criteria:

a) Insured by the medical system for the older adults aged 75 and over^†^.
b) Visited at least one of the venues where our additional health checkup tests were conducted in fiscal year (FY) 2018 or FY2019.
c) Agreed to participate in the study.

^†^The medical system for the older adults aged 75 and over was established in 2008 for fiscal adjustment (16). This system insures either (i) individuals aged 75 years and older or (ii) individuals aged between 65 and 75 years who are certified as having a certain degree of disability (e.g., hearing, vision, limb disability; internal organ disease; or mental disorder) as defined by the certification criteria of the Ministry of Health, Labour and Welfare.

#### 2.2.2 Control groups (usual health checkup group and no health checkup group)

The participants in the control groups were adult residents of Kure City aged 75 and over, who were insured by the medical system for the older adults aged 75 and over from FY2018 to FY2019. They did not undergo our additional health checkup tests. Individuals who indicated their intention to decline participation by opting out were excluded.

### 2.3 Procedure

#### 2.3.1 Intervention group

The researchers visited the usual health checkup sites and explained those who were fill the inclusion criteria in the Intervention group about the purpose, the content, privacy protection, and the freedom to participate in the study after which their written informed consent was obtained. In addition to the usual health checkup tests, the participants underwent risk screening tests for seven diseases (Table 1). Additional health checkup tests included questionnaires, and physical examinations and blood tests. Trained nurses conducted questionnaire surveys and physical examinations. For the blood test, a small amount of blood was added to the usual blood draw. The time burden imposed on the participants comprised approximately 30 min for the usual health checkups and a further 10 min for the additional items. The additional items are shown in Table 1.

**Table 1:** Usual health checkup tests and additional screening tests used in this study.

Usual health checkup tests (regulated by law)	Additional screening tests for seven high-risk diseases^§^
Questionnaire: medical history, medication, symptoms, lifestyle	Questionnaire: cognitive function test (Mini-Cog), Kihon checklist Screening for: cerebral infarction, dementia, frailty (difficulties in IADL, decreased locomotor function, undernutrition, decreased oral function, being homebound, decreased cognitive function, depressed mood)
Physical measurements: height, weight, BMI, blood pressure	Physical measurements: pulse rate, respiratory rate, SpO_2_, motor function test (Timed Up and Go test) Screening for: cerebral infarction (arrhythmia), HF, pneumonia, COPD, femur fracture (decreased locomotor function)
Blood tests: triglycerides, HDL-cholesterol, LDL-cholesterol, AST, ALT, *γ*-GT, fasting blood glucose/ HbA1c Urine tests: sugar, protein, (occult blood, urinary creatinine, urinary sodium, estimated salt intake)^§§^	Blood tests: NT-proBNP, serum creatinine (calculate eGFR)^§§^, total protein, albumin, hemoglobin, hematocrit Screening for: HF, CRF/decreased renal function, malnutrition/frailty/wasting disease (related to pneumonia)
Physical examination by physician	–

^§Some results of annual health checkups were also evaluated.^

^§§Kure City already included this item into their annual usual health checkups.^

IADL, instrumental activities of daily living; BMI, body mass index; SpO_2,_ percutaneous oxygen saturation; HF, heart failure; CRF, chronic renal failure; COPD, chronic obstructive pulmonary disease; HDL, high-density lipoprotein; LDL, low-density lipoprotein; AST, aspartate aminotransferase; ALT, alanine transaminase; γ-GT, gamma-glutamyl transferase; HbA1c, glycated hemoglobin; NT-proBNP, N-terminal pro b-type natriuretic peptide; eGFR, estimated glomerular filtration rate.

After all health checkup results were obtained, educational leaflets to enhance the understanding of the results and relevant information on risk reduction and the early detection of abnormalities were mailed to the participants’ homes together with the results of the usual health checkups. Based on the results of the checkup, the following information was included: self-management methods to reduce the risk of developing diseases (e.g., salt reduction, smoking cessation, strength training), self-monitoring methods for pulse rate, introduction of specific local health promotion and care prevention services, and information on receiving medical examinations. Thereafter, all participants received one in-person or telephonic individual guidance session, followed by one follow-up phone call 1–2 months later.

In the individual guidance session, the results of the health checkups were explained to the participants, and they were educated, introduced to services, and recommended to undergo medical examinations according to their individual risks. Telephonic sessions were held with participants who could not physically attend the guidance session. At the follow-up phone call 1–2 months after the guidance session, the status of the participants’ medical examinations and self-management was checked, and additional education was provided as necessary. When nurses determined that long-term follow-up, local long-term care prevention services, or use of the public medical care welfare system were necessary, they referred the participants to a public health nurse of the community with their prior consent.

#### 2.3.2 Control groups (usual health checkup group and no health checkup group)

After enrolment of the Intervention group (after FY2020), we screened the control group by going back through the historical data of the insured adult residents of Kure City aged 75 and over, who were insured by the medical system for the older adults aged 75 and over from FY2018 to FY2019. Besides, an option to opt-out was implemented for the control groups. At the time, Hiroshima University made opt-out information publicly available on its website, and Kure City provided the data to Hiroshima University in accordance with the city’s privacy protection ordinance. Researchers excluded the participants in the Intervention group and individuals who indicated their intention to decline participation by opting out. Then, they were divided into the two control groups (Usual Health Checkup group and No Health Checkup group).

The Usual Health Checkup group was defined as those who received usual health checkups in FY2018 or FY2019 but not the intervention in this study. Their medical claims data tracked for 2 years, starting the year following that in which they underwent a usual health checkup provided by Kure City.

The No Health Checkup group was defined as those who did not receive usual health checkups provided by Kure City in FY2018 or FY2019. Their medical claims data tracked for 2 years starting in 2020 in which they did not receive a usual health checkup.

### 2.4 Measures

#### 2.4.1 Usual health checkup tests and additional tests

The usual health checkup and additional tests are listed in Table 1. At the time of commencing the study (FY2018), the health checkup tests for the late-stage older adults as stipulated in the Act on Securing Medical Care for the Older were: a questionnaire (medical history, medication, symptoms, lifestyle), physical measurements (height, weight, body mass index, blood pressure), blood tests (triglycerides, high-density lipoprotein (HDL) cholesterol, low-density lipoprotein (LDL) cholesterol, aspartate aminotransferase, alanine transaminase, gamma-glutamyl transferase, fasting blood sugar/glycated hemoglobin (HbA1c)), urine tests (sugar, protein), and a physical examination by a physician.

In addition, we added the following risk screening tests for the seven diseases (femur fracture, cerebral infarction, CRF, HF, dementia, Alzheimer’s disease, pneumonia, and COPD). Tests from usual health checkups that could be used for risk screening purposes for the seven diseases were used where relevant.

#### 2.4.1.1 Questionnaire

The Mini-Cog© and Kihon checklist were used to screen for cognitive decline secondary to dementia/Alzheimer’s disease and frailty, respectively.

The Mini-Cog© consists of two components: a delayed three-word recall test and a clock drawing test (17), with scores ranging from 0 to 5 and higher total scores indicating higher cognitive function. This tool has been evaluated for reliability and validity, and a score of ≤2 is considered indicative of dementia (18).

As frailty is a risk factor for care-needy, deteriorated conditions in older adults. Frailty is also a risk factor for falls and fall-related fractures (19) and is associated with chronic conditions (20). Therefore, we considered that it is desirable to identify and improve frailty in the older adults to prevent disease onset and deterioration. The Kihon Checklist is a self-administered questionnaire in which respondents answer “yes” or “no.” The questionnaire consists of 25 items in seven domains: difficulties in activities of daily living, decline in locomotor function, undernutrition, decline in oral function, being homebound, decline in cognitive function, and depressed mood. Each of the domains was evaluated at the cutoff values, and their reliability and validity were confirmed (21, 22).

#### 2.4.1.2 Physical measurements

Pulse rate/arrhythmia, respiratory rate, and percutaneous oxygen saturation were used to screen for the risk of cardiogenic cerebral infarction and deterioration of respiratory and cardiac functions due to HF, CRF, pneumonia, and COPD. The Timed Up and Go (TUG) test was used to screen for a decline in locomotor function, which increases the risk of femoral fracture and frailty. The TUG test was developed as an index of functional mobility (23). First, the time required for the participant to stand up from a sitting position on a chair approximately 46 cm high, walk 3 m, change direction by 180°, walk 3 m again, and subsequently return to sitting on the chair was recorded. The TUG test has been validated for reliability and validity and correlates with walking ability, balance ability, and activities of daily living (23).

#### 2.4.1.3 Blood tests

N-terminal pro b-type natriuretic peptide (NT-proBNP), serum creatinine [to calculate estimated glomerular filtration rate (eGFR)], total protein, albumin, hemoglobin, and hematocrit were used to screen for HF, CRF/decreased renal function, and malnutrition/frailty/wasting disease related to pneumonia.

#### 2.4.2 Outcomes

Both the primary and secondary outcomes were defined as the incidence of events during the 2 years of follow-up and the 4 years including the pre-enrollment period. The primary outcome was a composite of hospitalization for any of the seven diseases (femoral fracture, stroke, CRF, HF, dementia, pneumonia, and COPD). The secondary outcomes included the use of emergency and critical care and hemodialysis induction.

### 2.5 Data collection

For the analysis, the required data were extracted from the claim data provided by the medical insurer in Kure City. Data on age and sex were collected as basic participant characteristics. Since none of the Intervention group were under 75 years of age, the claim analysis also selected insured persons over 75 years of age. For the outcomes, we collected data on the frequency of events during the 4-year period—from pre-enrollment to the end of the follow-up period (1 year before, 1 year during, and 2 years after enrollment). For each hospitalization incidence, the hospitalization medical claims data with the name of the disease was collected. Diseases were defined according to the International Classification of Diseases Tenth Revision as follows: femur fracture (S72), cerebral infarction (I63), CRF (N18), HF (I50), dementia (G30, F00–F03), pneumonia (J15–18, J69), and COPD (J40–44). Emergency and critical care were considered to have been used if any of the following was indicated on the medical claims data: emergency room inpatient charge, intensive care unit inpatient charge, high-care unit inpatient charge, or stroke care unit inpatient charge. Hemodialysis induction was also collected from medical claims data.

### 2.6 Statistical analysis

First, we described the entire results of usual health checkup tests and additional tests in the Intervention group. The data collected are presented as means ± standard deviations or numbers of persons and percentages. Second, to compare basic attributes among the three groups (age and sex), a one-way analysis of variance or chi-square test was performed after checking the distribution of the data for normality. Third, the frequency of events for each group (hospitalization, emergency & critical care, and hemodialysis) was tabulated for the 4-year period from pre-enrollment to follow-up. Logistic regression analysis using the forced entry method was subsequently conducted to analyze the effect of the intervention during the 2-year follow-up period, with the incidence of hospitalization as the primary outcome, use of emergency and critical care and the introduction of hemodialysis as the secondary outcome. Regression analysis was adjusted for sex and age at enrolment, with group as the dependent variable. A regression analysis was performed to ensure that the variance inflation factor of all independent variables was <10.

Then, we calculated approximate cost of additional checkup items. The cost of blood tests is regulated by the Japanese medical insurance system. Preparation of the explanation form for health checkup results was calculated based on actual time spent and the usual salary of the nurses. After adding mailing fee for results, the cost per participant was calculated.

SPSS software version 27.0 (IBM Corp., Armonk, NY, USA) was used for analysis and the significance level was set at 5%.

## 3 Results

All 492 individuals who met the eligibility criteria for the Intervention group agreed to participate (consent rates 100%). However, with the exception of seven individuals, including two who had missing data and five who received checkups under different conditions of implementation, 485 individuals underwent risk screening for the seven diseases and received a subsequent explanation of the results and health guidance. An additional 31 patients were lost to follow-up after 2 years. Therefore, 454 individuals were included in the efficacy analysis.

As for the control groups, the Usual Health Checkup group included 1,067 of the 1,096 participants who met the eligibility criteria and were available for follow-up. The No Health Checkup group included 43,712 of the 54,132 individuals who met the eligibility criteria and were followed up.

### 3.1 Characteristics of the participants in the intervention group

The number of persons in the Intervention group among the 485 who underwent risk screening for the seven diseases was 303 (62.5%) in the 75–79 year age group, 139 (28.6%) in the 80–84 year age group, 35 (7.2%) in the 85–89 year age group, and 8 (1.6%) in the 90+ year age group, with a mean age of 79.2 ± 3.6 years. This study included 253 (52.2%) men.

The health checkup results of the participants in the Intervention group are shown in Table 2. There were deviations from normal values for all items. The largest proportion of deviations occurred for systolic blood pressure (*n* = 307, 63.3%), estimated salt intake (*n* = 286, 60.3%), and LDL cholesterol (*n* = 250, 51.5%). eGFR deviated in 200 participants (41.0%), NT-proBNP in 184 participants (37.9%), hemoglobin in 57 men (22.5%) and 53 women (22.8%), and pulse rate (including arrhythmia) in 84 participants (17.4%). Thirty-six participants (7.5%) scored <2 points on the Mini-Cog, and 44 (9.1%) performed the TUG test in >12 s.

**Table 2:** Health checkup results in the intervention group.

Items	*n*	Mean ± SD	Normal values	Number of participants deviating from normal values			
				Total *n* (%)		Lower (%)	Upper (%)
Blood tests							
Fasting triglycerides (mg/dL)	485	103 ± 49	30–149	76	(15.7)	(0.0)	(15.7)
HDL-cholesterol (mg/dL)	485	65 ± 17	≥ 40	18	(3.7)	(3.7)	–
LDL-cholesterol (mg/dL)	485	121 ± 30	60–119	250	(51.5)	(2.1)	(49.5)
AST (U/L)	485	24 ± 7	30 ≥	52	(10.3)	–	(10.3)
ALT (U/L)	485	18 ± 8	30 ≥	24	(4.9)	–	(4.9)
γ-GT (U/L)	485	27 ± 18	50 ≥	34	(7.0)	–	(7.0)
Fasting blood glucose (mg/dL)	398	99 ± 17	70–99	135	(33.9)	(0.0)	(33.9)
HbA1c (%)	480	5.6 ± 0.6	4.6–6	223	(46.5)	(0.5)	(46.5)
Uric acid (mg/dL)	485	5 ± 1	3.6–7	71	(14.6)	(4.3)	(10.3)
eGFR (mL/min/1.73 m^2^)	485	63 ± 14	60–	200	(41.0)	(41.0)	–
[Men] Hematocrit (g/dL)	253	43 ± 4	38.5–49	40	(15.8)	(12.3)	(3.5)
[Women] Hematocrit (g/dL)	232	40 ± 3	35.5–44	42	(18.1)	(7.3)	(10.8)
[Men] Hemoglobin (g/dL)	253	14 ± 1	13.1–16	57	(22.5)	(18.2)	(4.3)
[Women] Hemoglobin (g/dL)	232	13 ± 1	12.1–15	53	(22.8)	(13.8)	(9.0)
Total protein (g/dL)	485	7 ± 0	6.5–8.0	26	(5.4)	(3.9)	(1.5)
Albumin (g/dL)	485	4 ± 0	≥ 4	67	(13.8)	(13.8)	–
NT-proBNP (pg/mL)	485	176 ± 304	125 ≥	184	(37.9)	–	(37.9)
Urine test							
Estimated salt intake (g/day)^†^	478	8.6 ± 2.2	8 ≥	288	(60.3)	–	(60.3)
Physical measurements							
BMI	485	23 ± 3	18.8–25	160	(33.0)	(6.6)	(26.4)
Systolic blood pressure (mmHg)	485	135 ± 18	90–129	307	(63.3)	(0.0)	(63.3)
Diastolic blood pressure (mmHg)	485	74 ± 10	84 ≥	67	(13.6)	–	(13.8)
Pulse rate (beats/min)	484	69 ± 11	60–90	84	(17.4)	(13.8)	(3.6)
Arrhythmia, *n* (%)	482			58	(12.1)		
Respiratory rate (breaths/min)	484	17 ± 2.6	20 ≥	24	(5.0)	–	(5.0)
SpO_2_ (%)	484	97 ± 1	≥ 96	36	(7.4)	(7.4)	–
Motor function: timed up and go (s)	483	8.3 ± 2.6	11 ≥	44	(9.1)	–	(9.1)
Questionnaire							
Cognitive function: Mini-Cog (score)	483	4 ± 1	3–5	36	(7.5)	(7.5)	–
Questionnaire: Kihon checklist							
Difficulties in activities of daily living (range: 1–20)	482		9 ≥	9	(1.9)		(1.9)
Decline in locomotor function (range: 1–5) *n* (%)	482		2 ≥	82	(17.0)		(17.0)
Undernutrition (range: 1–2) *n* (%)	482		1 ≥	6	(1.2)		(1.2)
Decline in oral function (range: 1–3) *n* (%)	482		1 ≥	76	(15.8)		(15.8)
Being homebound^§^ *n* (%)	482		Not applicable	21	(4.4)		(4.4)
Depressive mood (range: 1–5) *n* (%)	482		1 ≥	136	(28.2)		(28.2)

^†^Original standard of Kure City, Hiroshima Prefecture (less than 6 g according to the Japanese Society of Hypertension).

^§Estimated salt intake is assessed independently by Kure City as a usual heath checkup item.^

Results include those of participants using medication.

BMI, body mass index; HDL, high-density lipoprotein; LDL, low-density lipoprotein; AST, aspartate aminotransferase; ALT, alanine transaminase; γ-GT, gamma-glutamyl transferase; HbA1c, glycated hemoglobin; eGFR, estimated glomerular filtration rate; NT-proBNP, N-terminal pro b-type natriuretic peptide; SpO_2_, percutaneous oxygen saturation.

^§Being homebound is defined as not going out more than once per week.^

Furthermore, regarding the Kihon Checklist, the largest proportion of deviations occurred for depressive mood (*n* = 136, 28.2%), decline in locomotor function (*n* = 82, 17.0%), and decline in oral function (*n* = 76, 15.8%).

### 3.2 Comparison of participant attributes between the intervention and control groups (usual health checkup group and no health checkup group)

Table 3 shows the results of the comparison of baseline participant attributes among the three groups. Significant differences were observed in age and sex (*p* < 0.001). The highest age (79.1 ± 6.1 years) was recorded in the No Health Checkup group. In terms of sex, the highest percentage of women (61.3%) was found in the No Health Checkup group, while the lowest percentage (48.5%) was found in the Intervention group.

**Table 3:** Comparison of participant attributes between the Intervention and Comparison groups.

	Intervention group (*n* = 454)	Usual Health Checkup group (*n* = 1,067)	No Health Checkup group (*n* = 43,712)	*p*-value
Age, years (mean ± SD)	78.1 ± 3.5	77.8 ± 3.4	79.1 ± 6.1	<0.001^§^
Sex, *n* (%)				
Male	234(51.5%)	468(43.9%)	16,925(38.7%)	<0.001^§§^
Female	220(48.5%)	599(56.1%)	26,787(61.3%)	

^§ANOVA; §§Chi-square test; SD, standard deviation.^

### 3.3 Efficacy of the intervention

Table 4 shows the number and percentage of persons in whom an outcome occurred during the 4-year period from pre-enrollment to follow-up. For all events, the No Health Checkup group had the highest rate of event incidence.

**Table 4:** Number of individuals in whom events occurred in the three groups.

Events	Intervention group (*n* = 454)						Usual health checkup group (*n* = 1,067)						No health checkup group (*n* = 43,712)					
	Pre-enrollment period (1 year)		Enrollment period (1 year)		Follow-up period (2 years)		Pre-enrollment period (1 year)		Enrollment period (1 year)		Follow-up period (2 years)		Pre-enrollment period (1 year)		Enrollment period (1 year)		Follow-up period (2 years)	
Hospitalization^§^																		
Any of the 7 conditions	10	(2.2%)	23	(5.1%)	35	(8.1%)	22	(2.1%)	26	(2.4%)	106	(9.9%)	3,122	(7.1%)	3,75	(8.6%)	7,304	(16.7%)
Femur fracture	0	(0.0%)	2	(0.4%)	3	(0.7%)	0	(0.0%)	1	(0.1%)	10	(0.9%)	408	(0.9%)	442	(1.0%)	972	(2.2%)
Cerebral infarction	2	(0.4%)	7	(1.5%)	13	(2.9%)	4	(0.4%)	5	(0.5%)	28	(2.6%)	617	(1.4%)	776	(1.8%)	1,650	(3.8%)
CRF	2	(0.4%)	3	(0.7%)	4	(0.9%)	3	(0.3%)	4	(0.4%)	11	(1.0%)	422	(1.0%)	656	(1.5%)	1,103	(2.5%)
HF	4	(0.9%)	10	(2.2%)	16	(3.5%)	14	(1.3%)	16	(0.5%)	59	(5.5%)	1,588	(3.6%)	2,026	(4.6%)	3,959	(9.1%)
Dementia	0	(0.0%)	2	(0.4%)	8	(1.8%)	2	(0.2%)	3	(0.3%)	23	(2.2%)	828	(1.9%)	1,300	(3.0%)	2,313	(5.3%)
Pneumonia	1	(0.2%)	4	(0.9%)	7	(1.5%)	4	(0.4%)	3	(0.3%)	14	(1.3%)	641	(1.5%)	1,035	(2.4%)	1,800	(4.1%)
COPD	1	(0.2%)	2	(0.4%)	7	(1.5%)	3	(0.3%)	4	(0.4%)	16	(1.5%)	444	(1.0%)	494	(1.1%)	1,225	(2.8%)
Emergency & Critical Care (inpatient)	1	(0.2%)	5	(1.1%)	9	(2.0%)	4	(0.4%)	9	(0.8%)	24	(2.2%)	525	(1.2%)	580	(1.3%)	1,368	(3.1%)
Hemodialysis	0	(0.0%)	0	(0.0%)	2	(0.4%)	0	(0.0%)	0	(0.0%)	0	(0.0%)	218	(0.5%)	244	(0.6%)	337	(0.8%)

^§The name of the condition listed by the attending physician as the primary cause of hospitalization was recorded.^

CRF chronic renal failure, HF heart failure, COPD chronic obstructive pulmonary disease.

As a depiction of the intervention’s efficacy, Table 5 shows the results of the logistic regression analysis using the incidence of outcomes during the 2-year follow-up period as the objective variable. Hospitalization due to any of the seven diseases occurred significantly more frequently in the No Health Checkup group (odds ratio (OR) = 1.955, *p* < 0.001). HF was the most frequent cause of hospitalization among the three groups (Table 4), and the Intervention group had the lowest frequency of hospitalization due to femoral fracture (0.7%), CRF (0.9%), HF (3.5%), dementia (1.8%), and COPD (1.5%). In contrast, the No Health Checkup group had a higher frequency of hospitalization for all seven diseases, as well as for HF (9.1%), dementia (5.3%), pneumonia (4.1%), and stroke (3.8%). The use of emergency and critical care (inpatient) (the secondary outcome) did not significantly differ between the groups (OR = 0.199 and 1.955, *p* = 0.647 and 0.199 for the Usual Health Checkup and No Health Checkup groups, respectively). The difference between the groups was also not significant in terms of dialysis induction (OR = 0.000 and 1.930, *p* = 0.990 and 0.355 for the Usual Health Checkup group and the No Health Checkup group, respectively).

**Table 5:** Factors associated with outcome measures in the 2-year follow-up period after enrollment.

Factors	OR (95% CI)		*p*-value
Primary outcome: hospitalization due to any of the 7 diseases			
Group			
Intervention group	Reference		
Usual Health Checkup group	1.312	(0.884–1.947)	0.178
No Health Checkups group	1.965	(1.399–2.762)	< 0.001
Sex & Age			
Male	Reference		
Female	0.781	(0.740–0.824)	< 0.001
Age	1.113	(1.109–1.118)	< 0.001
Secondary outcomes: emergency & critical care (inpatient) use			
Group			
Intervention group	Reference		
Usual Health Checkup group	1.199	(0.552–2.602)	0.647
No Health Checkup group	1.545	(0.796–2.999)	0.199
Sex & Age			
Male	Reference		
Female	0.629	(0.564–0.701)	< 0.001
Age	1.064	(1.055–1.073)	< 0.001
Secondary outcomes: hemodialysis			
Group			
Intervention group	Reference		
Usual Health Checkup group	0.000	(0.000–0.000)	0.990
No Health Checkup group	1.930	(0.479–7.779)	0.355
Sex & Age			
Male	Reference		
Female	0.485	(0.390–0.604)	< 0.001
Age	0.997	(0.978–1.015)	0.718

OR, odds ratio; CI, confidence interval.

### 3.4 Cost of additional checkup items

Table 6 shows approximate cost of additional checkup items. Based on the actual cost used, the cost per participant was 3,682 JPY.

**Table 6:** Approximate cost of additional checkup items.

Items		Cost
Additional blood tests	NT pro-BNP	JPY 2,160 per person
	Total protein, albumin, hemoglobin, hematocrit	JPY 756 per person
Preparation of the explanation form for health checkup results	Labor costs related to the work	JPY 334 per person^§^ (JPY 162,000 per year)
Mailing fee for results	–	JPY 432 per person

NT-proBNP N-terminal pro b-type natriuretic peptide.

^§Cost was calculated on 485 participants who intervened in this study.^

## 4 Discussion

This study examined the rate of risks of onset and severity of seven diseases and the efficacy of proactive interventions based on the results of the community-based health checkups for the late-stage older adults in Japan. The newly added tests on the usual health checkups successfully screened at-risk individuals regarding seven diseases. A large proportion of the participants showed declines in renal and heart functions. Especially, it was important to be able to screen for the progression of frailty, malnutrition and cognitive decline in a situation where even the participants were not aware of it.

Since the data of Usual Health Checkup and No Health Checkup groups was collected on opt-out basis, we could not compare the detailed medical condition at baseline such as clinical data among the three groups. Although the potential for characteristics in the three groups could not be adjusted, the efficacy of intervention following the health check was evaluated by counting hospitalization and use of emergency and critical care with analysis of 2-year follow-up claim data. Although the results of the comparison were not significant among three groups, the Intervention group’s incidence rate of hospitalization due to any of the seven diseases was lower than that of the Usual Health Checkup group. Furthermore, the No Health Checkup group’s incidence of hospitalization was higher than the Intervention and Usual Health Checkup group. These findings might show screening for improvable risks and proactive interventions including individual self-management education, introduction to services and recommendation to undergo medical examinations are useful for preventing unplanned hospitalization of late-stage older adults. In addition, cost of additional checkup items would be acceptable, as it might contribute to early detection of the risk of expensive hospitalization.

### 4.1 Risk of development and exacerbation of diseases frequent in adults aged over 75 years

First, in the Intervention group, screening for the risk of developing or worsening femur fracture, cerebral infarction, CRF, HF, dementia, pneumonia, and COPD showed that more than half of the participants had high systolic blood pressure, LDL cholesterol levels, and estimated salt intake. Nearly half of the participants also deviated from the reference values for HbA1c, eGFR, NT-proBNP and fasting blood glucose levels, which are risk factors for cardiovascular diseases such as HF, ischemic heart disease, and stroke (24). Cardiovascular disease can lead to hospitalization and is the leading cause of death in patients aged over 75 years (25). In addition, patients with cardiovascular disease are often reported to have depressive tendencies, anxiety, and fear of activities of daily living (26, 27). Worsening mental health conditions and limitations in activities of daily living may lead to frailty and a need for nursing care (6). Therefore, early identification of older adults at risk of developing cardiovascular disease and interventions aimed at preventing severe disease will ensure disease control and contribute to the well-being and maintenance of independent living in these patients. Of the additionally screened items in this study, NT-proBNP showed the highest percentage of deviation from the reference value. NT-proBNP, a specific biomarker of HF, is associated with aging (28)—the values of as many as 40% of the Intervention group exceeded the reference values. The incidence of HF in Japan is reported to be 34% (29), which is similar to the proportion of individuals with high NT-proBNP levels in the Intervention group in this study. Therefore, the early identification of HF risk using this marker in adults aged over 75 years is clinically significant.

Second, cognitive function tests were conducted for dementia, which is estimated to have a prevalence of more than 10% in those aged 75 years and older in Japan (30). We found that 7.5% of the assessed patients scored below the cutoff value for suspected dementia. Cognitive decline among older adults occurs relatively slowly. Therefore, it is difficult for the older adults and their family members to notice changes. In some cases, dementia is diagnosed only when it progresses and interferes with daily life. Therefore, diagnosing dementia at an early stage, implementing appropriate disease management strategies, and creating a supportive environment will slow the progression of the disease and allow patients and their families to adjust their lives while accepting the changes.

Third, regarding the Kihon Checklist, 1.2 to 28.2% of the respondents were applicable each domain. In addition to changes in physiological and physical functions associated with aging (31, 32), many older adults have multiple diseases and conditions (33–35). Previous studies explained that their psychosomatic factors and social factors, including their connection to society, are interrelated (36). When assessing the health status of the older adults in health checkups, it is important to consider and conduct individualized interventions based on a multifaceted evaluation of the screening items added in this study and usual health checkup items.

### 4.2 Efficacy of proactive interventions based on the results of health checkups

The intervention group had fewer hospitalizations due to any of the seven diseases than did the Usual Health Checkup group, even though it was not statistically significant. When compared with the No Health Checkup group, it was statistically significant. This can be attributed to two factors. First, the nurses intervened according to the patients’ risk characteristics, and provided individualized education and referred them to clinics for further examination. Furthermore, when nurses determined that the participants needed long-term follow-up, local long-term care prevention services, or public healthcare and welfare services, they referred them to public health nurses of the municipal government. Individualized care coordination and monitoring for those at a high health risk were reported to be useful in previous studies (37–39). Second, nurses’ scientific-based explanations of health checkup results, motivation, and support for behavior change might be effective. Older adults often do not recognize their symptoms (40, 41), making it difficult for them to recognize the onset or severity of disease. In addition, even if they notice changes in their physical condition or memory ability, they may consider it merely being age-related and may not act to seek medical care. When explaining the results of the health checkups in this study, majority of participants were unaware of changes in their physical or cognitive deterioration or did not feel the need for medical examinations or self-management. The nurses’ easy explanations of the participants’ physical and cognitive condition, based on evidence-based clinical guidelines and other scientific evidence, may have helped the participants understand and convince them to change their behavior.

Moreover, during the 2-year follow-up period after enrollment, HF was found the most common cause of hospitalization in all three groups. Among them, the Intervention group showed the lowest percentage of hospitalizations secondary to HF. The nurses provided education on salt reduction and encouraged participants to comply medical treatment for hypertension, which is a strong risk factor for the development and progression of HF. In a previous study, >75% of patients with chronic HF who were hospitalized for acute exacerbations reported some form of inappropriate self-management (e.g., lack of regular hospital/clinic visits, excessive salt intake, lack of self-monitoring of weight) (42). For older adults with HF who require long-term self-management and medical care after disease onset, it is advisable for healthcare providers to regularly assess self-management behaviors, disease control status, self-management burden and anxiety, and changes in cognitive function, and adjust care as needed.

It should be noted that the background factors of the group that did not undergo medical checkups were not analyzed in this study. A survey on the characteristics of individuals who did not receive health checkups reported the following reasons: hassle, beliefs about health (43, 44), and lack of interest in health (45). In addition to these reasons, it is possible that a certain number of those who did not undergo health checkups in this study were in poor physical or mental condition at the time of enrollment, such as being hospitalized, living in a nursing home, or requiring significant assistance for transportation. It could be also assumed that some people who were already under medical treatment and considered the checkup unnecessary. Future studies verifying the effectiveness of the program should consider these background factors. In Japan, approximately 50% of the financial resources for medical insurance for adults aged 75 years and older are covered by public funds from national and local governments. Therefore, we believe that cost-effectiveness can be achieved if the onset of events is suppressed in relation to the additional cost of the new tests added to the health checkups (Table 6). The cost per hospitalization for Japanese adults aged 75 and older is reported approximately 62,000 JPY (46). Considering the additional costs of this study, cost effectiveness of screening for improvable risks and proactive interventions is significant. Although Japan has a high rate of outpatient visits among the Organization for Economic Co-operation and Development (OECD) countries (47), comprehensive risk screening is not performed during these visits. Therefore, we believe that it is necessary to add new health checkup tests for risk screening according to the characteristics of older adults in the late-stage of life. Regarding generalization in other municipalities, the added data collection through questionnaires and physical examinations are feasible for the general medical professionals. Health guidance includes comprehensive risk assessment, self-management education, introduction of formal and informal services. Therefore, it should be conducted by nurses/public health nurses with expertise. In the future, the nurse training conducted in this study need to be disseminated.

### 4.3 Limitations

The study has certain limitations. First, the population analyzed did not include those who died. Second, this study was limited to health checkup sites in a single municipality. The study included physical examinations, including the TUG test. This test requires a large space, which limited the number of health checkup sites at which the study could be conducted. Furthermore, in 2018 (the first year of the study), Kure City was hit by a major disaster (torrential rain and landslides), which hampered us to have health checkups in some sites. Therefore, the number of participants was lower than we have planned. Third, older adults who received health checkups usually have a high level of concern in their health and better physical and mental functions that enabled them to visit the site. Finally, this study did not include an analysis of the background factors for the incidence of outcome diseases and events, such as pre-existing medical conditions and the use of medication. Based on the results of health checkup, trained nurses confirmed the participants’ risk factors and educated them self-management methods. Participants had several multiple individual risk factors and common background factors could not be analyzed.

To measure effectiveness, it would need to be implemented in multiple municipalities and for a much larger population. The further study should conduct analyses that include mortality data and data on participants’ health status at the time of enrollment.

## 5 Conclusion

In this study, in addition to the usual government-mandated health checkup items, we conducted risk screening tests for the onset of seven diseases associated with the leading hospitalization costs in adults aged 75 years and older. Accordingly, individualized education and recommendations for medical examinations were provided to reduce risk, and referrals were made to public health nurses when necessary. The Intervention group had fewer hospitalizations due to any of the seven diseases than did the Usual Health Checkup and No Health Checkup groups. Individualized care coordination based on the results of health screening based on age-related changes could improve health outcomes. The national and municipal governments may need to add the screening tests of age-related seven diseases to the health checkup and consider preventive measures.

In future, the characteristics of participants who did not undergo health screening should be investigated to determine intervention strategies that meet their needs.

## Data Availability

The datasets presented in this article are not readily available because this study was conducted as the insurer’s project (Kure City) in accordance with the personal information protection ordinance.
